# Supplementary material for: A cost comparison of various hourly-reliable and net-zero hydrogen production pathways in the United States
Source: Nat Commun. 2023 Nov 15;14:7391. doi: 10.1038/s41467-023-43137-x (PMC10651927; doi:10.1038/s41467-023-43137-x)
Supplement: Supplementary file 1 — Supplementary Information [file 41467_2023_43137_MOESM1_ESM.pdf]

Supporting Information

A Cost Comparison of Various Hourly-Reliable and Net-Zero Hydrogen Production Pathways in the United States

Justin M. Bracci, Evan D. Sherwin, Naomi L. Boness, and Adam R. Brandt

Table of Contents

Supporting Information 1

1. Electricity-Based Hydrogen Production Mathematical Approach 4
2. Fossil-Based Hydrogen Production Mathematical Approach 9
3. Electricity-Based Hydrogen Production Configuration and Data Table 10
4. Fossil-Based Hydrogen Production Configuration and Data Tables 13
5. Electricity-Based Hydrogen Production Parameter Sensitivity Analysis 15
6. Fossil-Based Hydrogen Production Parameter Sensitivity Analysis 17
7. Next-Decade LCOH Input Parameter Sample Distributions for Error Bar Analysis 19
8. Next Decade Technology Hydrogen Production Pathway Emissions and LCOH Figures 21
9. Electricity-Based Hydrogen Production Pathway Operations Figures 23
10. LCOH Figures with Current and Mid-Century Timeframes 33
11. Inflation Reduction Act Analysis with Current Technology Timeframe 35

References 36

Tables and Figures

Table S.1: Electricity-based hydrogen production model input parameters for current, next decade, and mid-century timeframes.. 10
Table S.2: Embodied emissions estimates for various electricity generating technologies. 11
Table S.3: Fossil-based hydrogen production model input parameters. 13
Table S.4: Fossil-based hydrogen production model input parameters that change for current, next decade, and mid-century timeframes. 14
Table S.5: LCOH sensitivities explored for next decade electricity-based hydrogen production. 15
Table S.6: PV/Storage/Grid* LCOH sensitivity analysis with next decade technology 15
Figure S.1: PV/Storage/Grid* pathway spider plots with sensitivity results for 7 key input parameters 16
Table S.7: LCOH sensitivities explored for next decade fossil-based hydrogen production via ATR-CCS 17
Table S.8: Hourly reliable (ATR with process CO_2 capture) LCOH sensitivity analysis with next decade technology 17
Figure S.2: ATR-CCS pathway spider plots with sensitivity results for 7 key input parameters 18
Table S.9: Summary data table containing sample distributions for each input parameter 19
Figure S.3: Next decade GHG emissions of solar PV-based hydrogen production under various levels of reliability. 21
Figure S.4: Next-decade GHG emissions of various fossil-based hydrogen production pathways 21
Figure S.5: Levelized cost of hourly-reliable net-zero hydrogen produced from fossil-based pathways. 22
Table S.10: Next-decade electricity-based system component sizing and electricity utilization for all pathways 23
Figure S.6: Next decade electricity utilization for various levels of hydrogen production reliability pathways 24
Figure S.7: Next decade electricity utilization for hourly production reliability pathways 24
Figure S.8: Daily electricity utilization for the yearly reliable solar PV hydrogen production pathway in Sacramento, California 25
Figure S.9: Daily electricity utilization for the hourly reliable solar PV with storage hydrogen production pathway in Sacramento, California 25
Figure S.10: Daily electricity utilization for the hourly reliable solar PV, storage, and grid connected hydrogen production pathway in Sacramento, California 26
Figure S.11: Daily electricity utilization for the hourly reliable solar PV, storage, and limited grid connected hydrogen production pathway in Sacramento, California 26
Figure S.12: Hourly electricity utilization for the yearly reliable solar PV hydrogen production pathway for a week in the month of May in Sacramento, California 27
Figure S.13: Hourly electricity utilization for the hourly reliable solar PV and storage hydrogen production pathway for a week in the month of May in Sacramento, California 27
Figure S.14: Hourly electricity utilization for the hourly reliable solar PV, storage, and grid connected hydrogen production pathway for a week in the month of May in Sacramento, California 28
Figure S.15: Hourly electricity utilization for the hourly reliable solar PV, storage, and limited grid connected hydrogen production pathway for a week in the month of May in Sacramento, California 28
Figure S.16: Hourly hydrogen delivery for yearly reliable solar PV hydrogen production for a week in the month of May in Sacramento, California 29
Figure S.17: Hourly hydrogen delivery for hourly reliable solar PV and storage hydrogen production pathway for a week in the month of May in Sacramento, California 29
Figure S.18: Hourly hydrogen delivery for hourly reliable solar PV, storage, and grid connected hydrogen production pathway for a week in the month of May in Sacramento, California. 30

Figure S.19: Hourly hydrogen delivery for hourly reliable solar PV, storage, and limited grid connected hydrogen production pathway for a week in the month of May in Sacramento, California......................30
Figure S.20: Hourly hydrogen storage for hourly reliable solar PV and storage hydrogen production pathway for a week in the month of May in Sacramento, California. 31
Figure S.21: Hourly hydrogen storage for hourly reliable solar PV, storage, and grid connected hydrogen production pathway for a week in the month of May in Sacramento, California. 31
Figure S.22: Hourly hydrogen storage for hourly reliable solar PV, storage, and limited grid connected hydrogen production pathway for a week in the month of May in Sacramento, California......................32
Figure S.23: Net-zero hydrogen production cost comparison between all electricity-based production pathways looking at all timeframes and locations..33
Figure S.24: Hourly reliable, net-zero hydrogen production cost comparison between electricity-based and fossil-based production pathways looking at all timeframes and locations 34
Figure S.25: Current technology IRA emission and cost analysis 35

1. Electricity-Based Hydrogen Production Mathematical Approach

This section details the decision variables, constraints, and the objective function used for the electricity-based hydrogen production model.

Decision Variables:
S_B - Solar size (capacity of solar farm to build – kW_e)
E_B - Electrolyzer size (capacity of electrolyzer – kW_e)
T_B – Hydrogen storage tank size (kg_{H2})
B_B – Battery storage size (kWh_e)
L_A – Land required for PV facility (acres)
G_B – Grid connection size (kW_e)
E_U – Electricity used each hour of the year (kWh_e)
E_N – Electricity curtailed each hour of the year (kWh_e)
H_{UD} – Hydrogen used directly from electrolyzer each hour of the year (kg_{H2})
H_S – Hydrogen put into storage each hour of the year (kg_{H2})
H_T – Hydrogen taken and used from storage each hour of the year (kg_{H2})
H_{IS} – Hydrogen in storage each hour of the year (kg_{H2})
E_S – Electricity put into storage each hour of the year (kWh_e)
E_T – Electricity taken from storage each hour of the year (kWh_e)
E_{IS} – Electricity in storage each hour of the year (kWh_e)
E_G – Electricity taken from the grid each hour of the year (kWh_e)
C_A – Annual CO_2-equivalent emissions (kg_{CO2e})
W_{CA} – Annual water costs
R_E – Electrolyzer ramping (Change in kW_e between each hour of the year)
R_B – Battery storage ramping (Change in kWh_e stored between each hour of the year)
R_T – Hydrogen storage ramping (Change in kg_{H2} stored between each hour of the year)

Constraints:

Electricity Balance:

$$\forall i \in i = 1 \dots n: \ (CF_S)_i * (S_B) + (E_T)_i + m * (E_G)_i = (E_U)_i + (E_N)_i \qquad \text{Eq. \#1}$$

where:

- i = each hour of a year
- n = total number of hours in a year
- CF_S = solar PV capacity factor each hour of the year
- m = 0 or 1, depending on if a grid connection is considered

Electrolyzer Capacity Constraint:

$$\forall i \in i = 1 \dots n: \ (E_U)_i - (H_S)_i * (C_E) - (E_S)_i \leq (E_B) \qquad \text{Eq. \#2}$$

where:

- C_E = energy requirement for hydrogen compression (kWh_e/kg H_2)

Electricity and Hydrogen Energy Balance:

$$\forall i \in i = 1 \ldots n: \ (E_U)_i = \ (H_{UD})_i * (E_E) \ + (H_S)_i * \left((E_E) + \ (C_E)\right) + (E_S)_i \qquad \text{Eq. \#3}$$

where:

- E_E = electricity input requirement for hydrogen production from electrolysis (kWh_e/kg H_2)

Hydrogen Delivery Reliability Constraints:

$$\forall \ i \in i = 1 \ldots n: \ (H_{UD})_i + (H_T)_i = (\frac{P_E}{n}) \quad \text{with hourly hydrogen delivery constraint} \qquad \text{Eq. \#4}$$

$$\forall \ a \in a = 1 \ldots x: \ (H_{UD})_a + (H_T)_a = (\frac{P_E}{x}) \quad \text{with daily hydrogen delivery constraint} \qquad \text{Eq. \#5}$$

$$\forall \ b \in b = 1 \ldots y: \ (H_{UD})_b + (H_T)_b = (\frac{P_E}{y}) \quad \text{with monthly hydrogen delivery constraint} \qquad \text{Eq. \#6}$$

$$\forall \ c \in c = 1 \ldots z: \ (H_{UD})_c + (H_T)_c = (\frac{P_E}{z}) \quad \text{with yearly hydrogen delivery constraint} \qquad \text{Eq. \#7}$$

with:

$$\forall \ a \in a = 1 \ldots x: (H_{UD})_a \ , (H_T)_a = \sum_{i = (a-1)*\left(\frac{n}{x}\right)+1}^{a*\left(\frac{n}{x}\right)} (H_{UD})_i \ , (H_T)_i \qquad \text{Eq. \#8}$$

$$\forall \ b \in b = 1 \ldots y: (H_{UD})_b \ , (H_T)_b = \sum_{i = (b-1)*\left(\frac{n}{y}\right)+1}^{b*\left(\frac{n}{y}\right)} (H_{UD})_i \ , (H_T)_i \qquad \text{Eq. \#9}$$

$$\forall \ c \in c = 1 \ldots z: (H_{UD})_c \ , (H_T)_c = \sum_{i = (c-1)*\left(\frac{n}{z}\right)+1}^{c*\left(\frac{n}{z}\right)} (H_{UD})_i \ , (H_T)_i \qquad \text{Eq. \#10}$$

where:

- i = each hour per year, n = total hours per year = 8760
- x = each day per year, a = total days per year = 365
- y = each month per year, b = total months per year = 12
- z = each year per year, c = total years per year = 1
- P_E = yearly hydrogen production rate for electricity-based production (kg H_2 per year)

Grid Connection Capacity Constraints:

$$\forall i \in i = 1 \ldots n: \ (E_G)_i \leq \ (G_B) \qquad \text{Eq. \#11}$$

$$\forall i \in i = 1 \ldots n: \ (E_N)_i \leq \ (G_B) \qquad \text{Eq. \#12}$$

Grid Use Allowance:

$$\sum_{i=1}^{n} (E_G)_i \leq G_A * \sum_{i=1}^{n} ((E_U)_i + (E_N)_i) \qquad \text{Eq. \#13}$$

where:

- G_A = fraction of total electricity at hydrogen production facility that can be from the grid. This is set to 1 in the hourly *PV/Storage/Grid** pathway and set to 0.1 in the hourly *PV/Storage/Grid*** pathway.

Energy Storage Balance:

$$\forall i \in i = 1 \dots n\colon\ (H_{IS})_{i+1} - (H_{IS})_i = \eta_T * (H_S)_i - (H_T)_i \qquad \text{Eq. \#14}$$

$$\forall i \in i = 1 \dots n\colon\ (E_{IS})_{i+1} - (E_{IS})_i = \eta_B * (E_S)_i - (E_T)_i \qquad \text{Eq. \#15}$$

where:

- η_B = battery storage efficiency
- η_T = hydrogen tank storage efficiency

$$(H_{IS})_1 = (H_{IS})_n \qquad \text{Eq. \#16}$$

$$(E_{IS})_1 = (E_{IS})_n \qquad \text{Eq. \#17}$$

Energy Storage Capacity Constraints:

$$\forall i \in i = 1 \dots n\colon\ (H_{IS})_i <= (T_B) \qquad \text{Eq. \#18}$$
$$\forall i \in i = 1 \dots n\colon\ (H_S)_i <= (T_B) \qquad \text{Eq. \#19}$$
$$\forall i \in i = 1 \dots n\colon\ (H_T)_i <= (T_B) \qquad \text{Eq. \#20}$$

$$\forall i \in i = 1 \dots n\colon\ (E_{IS})_i <= (B_B) \qquad \text{Eq. \#21}$$
$$\forall i \in i = 1 \dots n\colon\ (E_S)_i <= (B_B) \qquad \text{Eq. \#22}$$
$$\forall i \in i = 1 \dots n\colon\ (E_T)_i <= (B_B) \qquad \text{Eq. \#23}$$

Land Area Calculation:

$$(L_A) = \left(\frac{S_B}{1000\frac{kW}{MW}}\right) * A_S \qquad \text{Eq. \#24}$$

where:

- A_S = Land area required per MW of solar PV installed (acres/MW)

Annual Emission Calculation:

$$C_A = (C_S) * \sum_{i=1}^{n} (CF_S)_i * (S_B) + (m) * \sum_{i=1}^{n} (E_G)_i * (C_{G,pc})_i \qquad \text{Eq. \#25}$$

where:

- C_S = the life-cycle carbon intensity of solar PV (kg CO_2e / kWh_e)
- $C_{G,pc}$ = the operations-related grid emissions data for each hour of a year (kg CO_2e / kWh_e)

with:

$$\forall i \in i = 1 \dots n\text{: } (C_G)_i = (C_{G,pc})_i + \frac{\sum_{g=1}^{gt}(C_{em})_g * (E_{prod})_{i,g}}{\sum_{g=1}^{gt}(E_{prod})_{i,g}} \quad \text{Eq. \#26}$$

where:

- C_G = the life-cycle carbon intensity of the grid each hour of a year (kg CO_2e / kWh_e). Calculated outside of the optimization.
- g = electricity generation technology
- g_t = total number of electricity generating technologies in the grid portfolio
- C_{em} = the embodied emissions of each electricity generation technology (kg CO_2e / kWh_e)
- E_{prod} = electricity generated in each hour i, by each technology type g, to support grid demands (kWh_e)

Annual Water Cost Calculation:

$$W_{CA} = (W_C) * (P) * (W_U) \quad \text{Eq. \#27}$$

where:

- W_C = the cost of water (\$ / kg H_2O)
- W_U = the usage rate of water (kg H_2O / kg H_2 produced)

Ramping Constraints:

$$\forall i \in i = 1 \dots n - 1\text{: } (R_E)_i \geq (E_U)_i - (E_U)_{i+1} \quad \text{Eq. \#28}$$
$$\forall i \in i = 1 \dots n - 1\text{: } (R_E)_i \geq -((E_U)_i - (E_U)_{i+1}) \quad \text{Eq. \#29}$$

$$\forall i \in i = 1 \dots n - 1\text{: } (R_B)_i \geq (E_{IS})_i - (E_{IS})_{i+1} \quad \text{Eq. \#30}$$
$$\forall i \in i = 1 \dots n - 1\text{: } (R_B)_i \geq -((E_{IS})_i - (E_{IS})_{i+1}) \quad \text{Eq. \#31}$$

$$\forall i \in i = 1 \dots n - 1\text{: } (R_T)_i \geq (H_T)_i - (H_T)_{i+1} \quad \text{Eq. \#32}$$
$$\forall i \in i = 1 \dots n - 1\text{: } (R_T)_i \geq -((H_T)_i - (H_T)_{i+1}) \quad \text{Eq. \#33}$$

<u>Objective Function:</u>

Minimize the levelized cost of hydrogen produced (LCOH= f(x)). LCOH is equivalent to the annualized hydrogen production cost divided by the annual hydrogen production rate:

$min\text{: } f(x) =$

$$\frac{\begin{array}{c}[(CRF + S_{OM}) * S_B * S_C + (CRF + E_{OM}) * E_B * E_C \\ + (CRF + T_{OM}) * T_B * T_C + (CRF + B_{OM}) * B_B * B_C \\ + m * \sum_{i=1}^{n}((E_G)_i * (LMP)_i - (E_N)_i * (NSCR)_i) + W_{CA} \\ + m * (G_B * (D_C + I_C) * b + F_C \\ + m * (CRF + G_{OM}) * G_B * G_C + (L_A * L_C) + (C_A * M_C) \\ + \sum_{i=1}^{n-1}((R_E)_i * (R)_{C1} + (R_T)_i * (R)_{C2}) + \sum_{i=1}^{n-2}((R_B)_i * (R)_{C1})]\end{array}}{P_E} \quad \text{Eq. \#34}$$

where:

- CRF = capital recovery factor (% CAPEX/yr)
- S_{OM} = solar PV operation and maintenance cost (% CAPEX/yr)
- S_C = solar PV capital cost ($/kW$_e$)
- E_{OM} = electrolyzer operation and maintenance cost (% CAPEX/yr)
- E_C = electrolyzer capital cost ($/kW$_e$)
- T_{OM} = hydrogen storage tank operation and maintenance cost (% CAPEX/yr)
- T_C = hydrogen storage tank capital cost ($/kg$_{H2}$)
- B_{OM} = battery storage operation and maintenance cost (% CAPEX/yr)
- B_C = battery storage capital cost ($/kWh$_e$)
- LMP = hourly locational marginal electricity pricing ($/kWh$_e$)
- $NSCR$ = net surplus compensation rate each hour ($/kWh$_e$)
- D_C = grid demand charges ($/max kW$_e$/month)
- I_C = grid infrastructure charge ($/kW$_e$/month)
- F_C = fixed grid infrastructure charge ($/month)
- G_{OM} = grid connection operation and maintenance cost (% CAPEX/yr)
- G_C = grid connection capital cost ($/kW$_e$)
- L_C = cost for land lease ($/acre/yr)
- M_C = cost for CO_2 removal ($/kg CO_2 removed)
- R_{C1} = cost incurred due to electrical system ramping ($/change in kWh$_e$). Prevents unphysical ramping patterns and amounts to about $0.10/kg or less.
- R_{C2} = cost incurred due to hydrogen system ramping ($/change in kg H_2). Prevents unphysical ramping patterns and amounts to about $0.10/kg or less.

and:

$$CRF = \frac{WACC}{1-(1+WACC)^{-t}} \quad \text{Eq. \#35}$$

where:

- $WACC$ = weighted average cost of capital, also known as a discount rate (%)
- t = project life (years)

2. Fossil-Based Hydrogen Production Mathematical Approach

This section details the fossil-based hydrogen production computational model input variables and equations. The primary equation being the LCOH from fossil-derived hydrogen production.

$$LCOH_j = \frac{(CRF) * \left((C_R)_j + (C_{PC})_j + (C_{FC})_j + (C_{AS})_j\right) + (F_{OM})_j + (V_{OM})_j}{F_1} + (NG_C) * (HV_{NG}) * (I_{NG})_j + \left(E_{P,avg}\right) * (I_E)_j + (C_{CTS}) * (O_{CC})_j + (M_C) * (E_F)_j \quad \text{Eq. \#36}$$

and:

$$(E_f)_j = (E_D)_j + \left(C_{G,avg}\right) * (I_E)_j + (NG_L) * (I_{NG})_j * (GWP) + (NG_P) * (I_{NG})_j \quad \text{Eq. \#37}$$

where:

- j = the fossil-based production pathway we are exploring
- C_R = the capital cost of the reformer (\$/kW H_2)
- C_{PC} = the capital cost of process CO_2 capture (\$/kW H_2)
- C_{FC} = the capital cost of flue gas CO_2 capture (\$/kW H_2)
- C_{AS} = the capital cost of an air separation unit (\$/kW H_2)
- F_{OM} = the fixed operating cost of the facility (\$/kW/yr H_2)
- V_{OM} = the variable operating cost of the facility (\$/kW/yr H_2)
- F_1 = conversion factor from kW_{H2} to kg H_2 per year using lower heating value (263 [kg/yr]/kW)
- NG_C = natural gas cost (\$/MJ)
- HV_{NG} = the heating value of natural gas (52 MJ/kg)
- I_{NG} = natural gas input at the facility (kg CH_4/kg H_2)
- $E_{P,\ avg}$ = the average electricity price in the location considering locational marginal pricing and grid use charges (\$/kWh_e)
- I_E = electricity input at the facility (kWh_e/kg H_2)
- C_{CTS} = the cost for CO_2 transport and storage (\$/kg CO_2)
- O_{CC} = carbon captured at the facility (kg CO_2/kg H_2)
- M_C = the cost for carbon removal (\$/kg CO_2)
- E_F = all facility related and upstream carbon emissions (kg CO_2/kg H_2)
- E_D = direct facility carbon emissions (kg CO_2/kg H_2)
- $C_{G,avg}$ = the average carbon intensity of the grid in the location of interest (kg CO_2e/kWh_e)
- NG_L = the fraction of the natural gas input that leaks during natural gas production and processing (kg CH_4 leaked/kg CH_4 input)
- GWP = global warming potential of CH_4 relative to CO_2. Typically measured using a 20-year or 100-year timeframe. We use a 20-year timeframe in our base case.
- NG_P = carbon emission associated with natural gas production and processing (kg CO_2/kg CH_4)

3. Electricity-Based Hydrogen Production Configuration and Data Table

This section contains data tables of the primary inputs used to develop the electricity-based production model results. In addition to next-decade technology, Table S.1 also contains input data for current and mid-century technology assumptions. Cost data are drawn from sources with values in 2017 through 2021 dollars. We assume our results are in 2020 dollars without harmonizing input costs since they are all plus-or-minus 5% of the 2020 dollar value [1].

Table S.2 lists the embodied emissions assumptions we use for each electricity generator. The emissions data from NREL's Cambium datasets only include pre-combustion and combustion-related grid emissions. We adjust this data to include all life-cycle emissions of the electricity generating facilities that support the grid (see Annual Emission Constraint in Section #1).

*Table S.1: Electricity-based hydrogen production model input parameters for current, next decade, and mid-century timeframes. * Next decade values are used to generate model results shown in main text. ** Slightly less than full marginal prices are used to help improve solver convergence and to account for potential system losses.*

Parameter	Current Value	Next Decade Value *	Mid-Century Value	Units	Source
Hydrogen Supply	25	250	500	metric ton/day	Assumed
CA Solar PV Capacity Factor (hourly)	27.5	27.5	27.5	% average	[2]
TX Solar PV Capacity Factor (hourly)	29.6	29.6	29.6	% average	[2]
NY Solar PV Capacity Factor (hourly)	20.0	20.0	20.0	% average	[2]
Electrolysis Efficiency	60	65	70	%	[3]
H_2 Storage Efficiency	100	100	100	%	Assumed
Battery Storage Efficiency	80	85	90	%	[3]
Hydrogen Energy Content (Lower Heating Value)	33.3	33.3	33.3	kWh_{H2}/kg_{H2}	[4]
Compression Energy Input	1.2	1.2	1.1	kWh_e/kg_{H2}	[3]
Capital Cost of Electrolyzer (2020 dollars)	890	460	385	$\$/kW_e$ (100 MW system)	[3], [5], [6]
Capital Cost of Solar Farm (2020 dollars)	900	600	500	$\$/kW_e$ (100 MW system)	[3], [7], [8]
Capital Cost Grid Connection (2017 dollars)	340	180	45	$\$/kW_e$	[3]
Electrolyzer O&M (2020 dollars)	7.5	7.5	7.5	%/year CAPEX	[3], [5]
Solar O&M (2020 dollars)	2	2	2	%/year CAPEX	[8]
Grid Connect O&M (2017 dollars)	1	1	1	%/year CAPEX	[3]
Capital Cost H_2 Storage (2017 dollars)	830	500	200	$\$/kg_{H2}$	[3], [9]
Capital Cost Battery Storage (2020 dollars)	350	250	100	$\$/kWh_e$	[3], [8], [10]
H_2 Storage O&M (2017 dollars)	1	1	1	%/year CAPEX	[3]
Battery Storage O&M (2020 dollars)	2.5	2.5	2.5	%/year CAPEX	[8]
Project Life	25	25	25	years	[3]

WACC	10	8	5	%	[3]
Electrolyzer Cost Scaling Factor	0.95	0.95	0.95	N/A	Assumed
Solar PV Cost Scaling Factor	0.9	0.9	0.9	N/A	Assumed
Water Cost (2017 dollars)	1	1	1	$/ton	[3]
Water Usage	15	15	15	kg H_2O/kg H2	[11]
Solar Land Cost (2020 dollars)	750	750	750	$/acre/year	[12]
Land for Solar Farm	7.5	7.5	7.5	acres/MW	[13]
CA Grid Electricity Cost (hourly) (2021 dollars)	0.040	0.033	0.032	$/kWh$_e$ yearly average	[14]
TX Grid Electricity Cost (hourly) (2021 dollars)	0.028	0.023	0.021	$/kWh$_e$ yearly average	[14]
NY Grid Electricity Cost (hourly) (2021 dollars)	0.041	0.036	0.038	$/kWh$_e$ yearly average	[14]
Net Surplus Compensation Rate (2021 dollars)	99% of hourly electricity costs**			$/kWh$_e$	Assumed
CA Life-Cycle Grid Emissions (hourly)	0.20	0.077	0.025	kg CO_2/kWh$_e$ average	[14]–[16]
TX Life-Cycle Grid Emissions (hourly)	0.29	0.077	0.068	kg CO_2/kWh$_e$ average	[14]–[16]
NY Life-Cycle Grid Emissions (hourly)	0.23	0.038	0.013	kg CO_2/kWh$_e$ average	[14]–[16]
CA Grid Demand Charge (2017 dollars)	10	10	10	$/max kW$_e$/month	[17]
TX Grid Demand Charge (2017 dollars)	5	5	5	$/max kW$_e$/month	[17]
NY Grid Demand Charge (2017 dollars)	5	5	5	$/max kW$_e$/month	[17]
Solar PV Lifecycle Emissions	0.04	0.04	0.03	kg CO_2/kWh$_e$	[18]
CO_2 Removal Cost (2017 dollars)	600	200	100	$/metric ton CO_2 removed	[3], [19]

*Table S.2: Embodied emissions estimates for various electricity generating technologies. * Values are close enough to zero to have a negligible impact on results if included.*

Technology	Embodied Emissions (kg CO2$_e$/kWh$_e$)	Source
Utility-Scale Battery	40	[15], [16]
Biomass	0*	
Biomass w/CCS	0*	
Imports	0*	
Coal	0*	
Coal w/CCS	0*	
Concentrated Solar Power	30	
Distributed PV	40	
Gas Combined Cycle	0*	
Gas Combined Cycle w/CCS	0*	
Gas Combustion Turbine	0*	
Geothermal	40	
Hydropower	5	
Nuclear	0*	
Oil-Gas-Steam	0*	
Pumped Hydro Storage	5	

Utility-Scale PV	40	
Onshore Wind	10	
Offshore Wind	10	

4. Fossil-Based Hydrogen Production Configuration and Data Tables

This section contains data tables of the primary inputs used to develop the fossil-based production model results. In addition to next-decade technology, Table S.4 also contains input data that changes with current and mid-century technology assumptions. Cost data are drawn from sources with values in 2017 through 2021 dollars. We assume our results are in 2020 dollars without harmonizing input costs as they are all plus-or-minus 5% of the 2020 dollar value [1].

Table S.3: Fossil-based hydrogen production model input parameters. (1) Refers to an SMR with process CO_2 capture. (2) Refers to an SMR with process and flue gas CO_2 capture. (3) Refers to an ATR with process CO_2 capture.

Input Parameters	Production Method				Units	Source
	SMR	SMR-CCS (1)	SMR-CCS (2)	ATR-CCS (3)		
Capacity Factor	90%	90%	90%	90%	N/A	[20]
CO_2 Capture Percent	0%	56%	96%	95%	%	[20]
Baseline H_2 Production Capacity	483	483	483	660	metric ton/day	[20]
Next Decade Production Capacity	250	250	250	250	metric ton/day	N/A
Plant Life	30	30	30	30	years	[21]
Electricity Use	0.65	1.5	2.04	4	kWh_e/kg H_2 capacity	[20]
Natural Gas Use	3.53	3.58	3.75	3.52	kg CH4/kg H_2 capacity	[20]
Water Consumption	16	19	24	24	kg H_2O/kg H_2 capacity	[20]
Reformer/Other Capital Cost (2018 dollars)	549	576	809	604	$/kW H_2 capacity	[20]
Air Separation Unit Capital Cost (2018 dollars)	0	0	0	294	$/kW H_2 capacity	[20]
Process Capture Capital Cost (2018 dollars)	0	158	61	158	$/kW H_2 capacity	[20]
Flue Gas Capture Capital Cost (2018 dollars)	0	0	466	0	$/kW H_2 capacity	[20]
Fixed O&M (2018 dollars)	16	23	35	27	$/kW per year H_2 capacity	[20]
Other Variable O&M (2018 dollars)	10	14	23	17	$/kW per year H_2 capacity	[20]
CA Natural Gas Cost (2020 dollars)	6.5	6.5	6.5	6.5	$/MMTBU CH_4	[5], [22]
TX Natural Gas Cost (2020 dollars)	4	4	4	4	$/MMBTU CH_4	[5], [22]
NY Natural Gas Cost (2020 dollars)	5	5	5	5	$/MMBTU CH_4	[5], [22]
Natural Gas Energy Content (Heating Value)	52	52	52	52	MJ/kg CH_4	N/A
Discount Rate (WACC)	5%	5%	5%	5%	%	[20]
Cost Scaling Factor	0.6	0.6	0.6	0.6	N/A	[23]
Direct CO_2 Emissions	9.3	4.1	0.4	0.5	kg CO_2/kg H2	[20]
CO_2 Captured	0	5.2	10.1	8.6	kg CO_2/kg H2	[20]
Upstream Natural Gas Processing Carbon Intensity	0.3	0.3	0.3	0.3	kg CO_2/kg CH4	[24]
Grid Carbon Intensity	0.12	0.12	0.12	0.12	kg CO_2/kWh_e electricity	[25]

CO_2 Transport and Storage Costs (2020 dollars)	0	10	10	10	\$/metric ton CO_2 captured	[26]
CO_2 Removal Cost (2017 dollars)	200	200	200	200	\$/metric ton CO_2 removed	[3], [19]
Natural Gas GWP 100	30	30	30	30	kg CO_2e/kg CH_4	[27]
Natural Gas GWP 20	85	85	85	85	kg CO_2e/kg CH_4	[27], [28]

Table S.4: Fossil-based hydrogen production model input parameters that change for current, next decade, and mid-century timeframes.

Parameter	Current Value	Next Decade Value	Mid-Century Value	Units	Source
Hydrogen Supply	25	250	500	metric ton/day	Assumed
Capital Cost Grid Connection (2017 dollars)	340	180	45	\$/kW_e	[3]
Grid Electricity Cost (hourly) (2021 dollars)	same as in Table S.1 for each state			\$/kWh_e yearly average	[14]
Life-Cycle Grid Emissions (hourly)	same as in Table S.1 for each state			kg CO_2/kWh_e average	[14]–[16]
Natural Gas Processing Emissions	0.5	0.3	0.1	kg CO_2/kWh_e	[18]
CO_2 Removal Cost (2017 dollars)	600	200	100	\$/metric ton CO_2 captured	[3], [19]

5. Electricity-Based Hydrogen Production Parameter Sensitivity Analysis

This section contains the raw data tables for the electricity-based hydrogen production model sensitivity analysis. Sensitivity results shown in these raw data tables are for the hourly-reliable *PV/Storage/Grid** hydrogen production scenario. Spider plots with key parameter sensitivities are shown in Figure S.1. All low- and high-end input values are drawn from the current and mid-century assumptions, unless cited or stated otherwise in Table S.5.

Table S.5: LCOH sensitivities explored for next decade electricity-based hydrogen production. Grid electricity related parameter changes are only explored in the pathways with a grid connection.

Parameter	Parameter Change: Lower LCOH	Parameter Change: Higher LCOH
Grid Connection Capital Cost	$45/kW$_e$	$340/kW$_e$
Battery Storage Capital Cost	$100/kWh$_e$	$350/kWh$_e$
Surplus Compensation Rate	Same as listed in Table S.1	No compensation
H_2 Storage Capital Cost	$200/kg	$830/kg
Project Life	30 years<br>(+ 20% from base case)	20 years<br>(- 20% from base case)
Hourly Grid Electricity Prices	Cambium: Low Renewable Energy Cost Scenario [14]	Cambium: High Renewable Energy Cost Scenario [14]
Solar Life Cycle Emissions	0.00 kg CO_2/kWh$_e$<br>(Embodied emissions left out)	0.05 kg CO_2/kWh$_e$ [18]
Electrolyzer Efficiency	70%	60%
Discount Rate (WACC)	5%	10%
Grid Demand Charge	$5/kW$_e$ [17]	$30/kW$_e$ [17]
Electrolyzer Capital Cost	$340/kW$_e$	$915/kW$_e$
Solar Capital Cost	$400/kW$_e$	$950/kW$_e$
CO_2 Removal Cost	$100/ton CO_{2e}	$600/ton CO_{2e}

Table S.6: PV/Storage/Grid LCOH sensitivity analysis with next decade technology.*

Parameter	Lower LCOH			Higher LCOH		
	CA	NY	TX	CA	NY	TX
Grid Connection Capital Cost	$2.79	$2.28	$1.92	$2.98	$2.50	$2.14
Battery Storage Capital Cost	$2.68	$2.09	$1.73	$2.96	$2.48	$2.11
Surplus Compensation Rate	$2.88	$2.39	$2.02	$3.15	$2.39	$2.02
H2 Storage Capital Cost	$2.86	$2.34	$1.94	$2.91	$2.42	$2.07
Project Life	$2.85	$2.35	$1.98	$2.94	$2.45	$2.08
Average Annual Electricity Cost	$2.56	$2.19	$1.71	$3.10	$2.59	$2.26
Solar Life Cycle Emissions	$2.79	$2.35	$2.00	$2.90	$2.39	$2.04
Electrolyzer Efficiency	$2.69	$2.23	$1.89	$3.11	$2.58	$2.18
Discount Rate (WACC)	$2.68	$2.20	$1.80	$3.00	$2.48	$2.14
Average Annual Grid Carbon Intensity	$2.48	$2.39	$2.02	$4.38	$4.09	$3.69
Electrolyzer Capital Cost	$2.79	$2.29	$1.93	$3.42	$2.92	$2.59
Solar Capital Cost	$2.84	$2.37	$2.00	$2.92	$2.38	$2.03
CO_2 Removal Cost	$2.52	$2.19	$1.72	$4.40	$3.11	$3.13

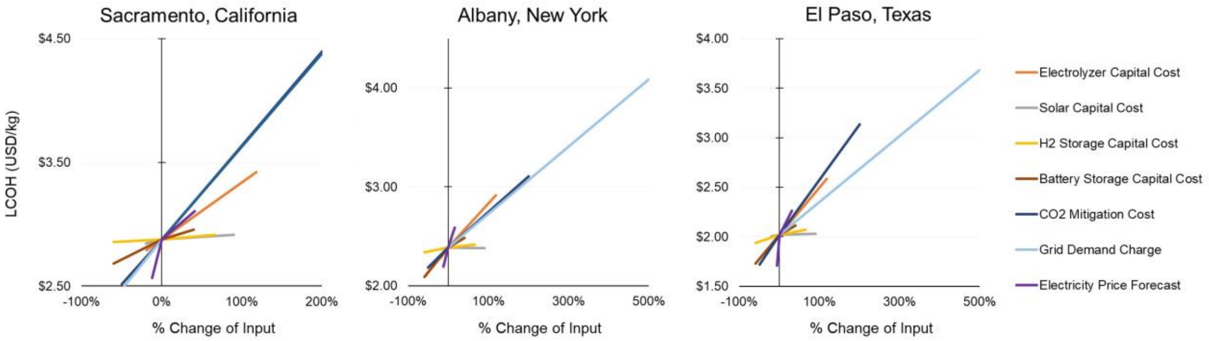

Figure S.1: PV/Storage/Grid pathway spider plots with sensitivity results for 7 key input parameters.*

6. Fossil-Based Hydrogen Production Parameter Sensitivity Analysis

This section contains the raw data tables for the fossil-based hydrogen production model sensitivity analysis. The sensitivity results shown in these raw data tables are for the *ATR-CSS* hydrogen production scenario. Spider plots with key parameter sensitivities are shown in Figure S.2. All low- and high-end input values are drawn from the current and mid-century assumptions, unless cited or stated otherwise in Table S.7.

Table S.7: LCOH sensitivities explored for next decade fossil-based hydrogen production via ATR-CCS.

Parameter	Parameter Change: Lower LCOH	Parameter Change: Higher LCOH
CO_2 Transport and Storage Cost	\$10/ton CO_2 [26]	\$20/ton CO_2 [26]
Economies of Scale Factor	0.8 [23]	0.55 [23]
Plant Life	40 years<br>(+ 33% from base case)	20 years<br>(- 33% from base case)
Grid Demand Charge	\$5/kW$_e$ [17]	\$30/kW$_e$ [17]
Hourly Grid Electricity Prices	Cambium: Low Renewable Energy Cost Scenario	Cambium: High Renewable Energy Cost Scenario
Natural Gas Processing Emissions	0.1 kg CO_2/kg CH_4 [24]	0.5 kg CO_2/kg CH_4 [24]
Discount Rate (WACC)	4% [21]<br>(20% less than base case)	10% [3]
GWP Timeframe	100-year [27]	20-year [27]
Facility Size	500 ton/day	25 ton/day
Natural Gas Price	\$2.84/GJ (\$3/MMBTU) [22]	\$14.22/GJ (\$15/MMBTU) [22], [29]
Natural Gas Leakage Rate (GWP20)	0%<br>(CH_4 releases are mitigated)	4% [30], [31]
CO_2 Removal Cost	\$100/ton CO_2	\$600/ton CO_2

Table S.8: Hourly reliable (ATR with process CO_2 capture) LCOH sensitivity analysis with next decade technology.

Parameter	Lower LCOH			Higher LCOH		
	CA	NY	TX	CA	NY	TX
CO_2 Transport and Storage Cost	\$3.28	\$2.97	\$2.78	\$3.37	\$3.06	\$2.86
Economies of Scale Factor	\$3.18	\$2.88	\$2.68	\$3.31	\$3.00	\$2.80
Plant Life	\$3.24	\$2.93	\$2.74	\$3.37	\$3.06	\$2.87
Grid Demand Charge	\$3.25	\$2.97	\$2.78	\$3.39	\$3.11	\$2.91
Grid Electricity Cost	\$3.25	\$2.96	\$2.75	\$3.33	\$2.99	\$2.85
Natural Gas Processing Emissions	\$3.14	\$2.83	\$2.64	\$3.42	\$3.11	\$2.92
Discount Rate (WACC)	\$3.24	\$2.93	\$2.73	\$3.53	\$3.22	\$3.02
GWP Timeframe	\$2.70	\$2.39	\$2.20	\$3.28	\$2.97	\$2.78
Facility Size	\$3.15	\$2.84	\$2.65	\$4.09	\$3.78	\$3.59
Natural Gas Price	\$2.67	\$2.63	\$2.60	\$4.75	\$4.71	\$4.69
Natural Gas Leakage Rate (GWP20)	\$2.38	\$2.07	\$1.88	\$4.78	\$4.47	\$4.27
CO_2 Removal Cost	\$2.64	\$2.35	\$2.14	\$5.82	\$5.45	\$5.32

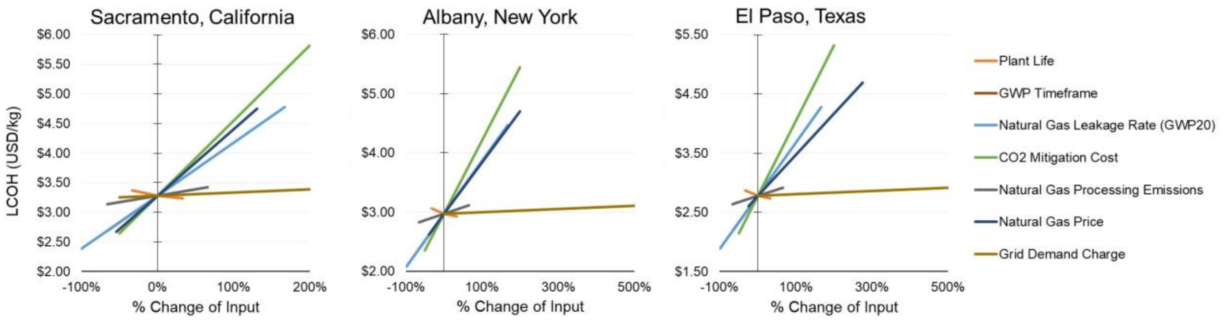

Figure S.2: ATR-CCS pathway spider plots with sensitivity results for 7 key input parameters.

7. Next-Decade LCOH Input Parameter Sample Distributions for Error Bar Analysis

The raw data table (Table S.9) in this section contains details on the distributions chosen for each input parameter. Monte Carlo simulation is performed using the information in this raw data table to generate the errors bars in main text Figure 4. For most input parameters, triangular distributions are chosen with lower and upper bounds of the distributions set as the current and mid-century technology options from Tables S.1 and S.4. All other low- and high-end bound in Table S.9 are drawn from Tables S.5 and S.7 or are cited in Table S.9. Notably, we sample from three different 2035 grid pricing and emission scenarios for each state. These scenarios are pulled from NREL's Cambium database and are listed as: Mid Case (base), Mid Case with Low Renewable Energy Costs, and Mid Case with High Renewable Energy Costs.

Table S.9: Summary data table containing sample distributions for each input parameter. These distributions are used to generate error bars on next-decade LCOH figures through Monte Carlo simulation.

Parameters		Distribution Type	Low Value	High Value	Base Value	Units
Electricity-Based H_2	Electrolyzer Efficiency	Triangular	60	70	65	%
	Electrolyzer Capital Cost	Triangular	340	915	340	$/kW$_e$
	Solar Capital Cost	Triangular	400	950	400	$/kW$_e$
	H_2 Storage Capital Cost	Triangular	200	830	500	$/kg
	Battery Storage Capital Cost	Triangular	100	350	250	$/kWh$_e$
	Solar Land Cost	Triangular	500 [12]	1250 [12]	750 [12]	$/acre/yr
	Project Life	Triangular	20	30	25	Years
	WACC	Triangular	5	10	8	%
	Solar Life Cycle Emissions	Triangular	0	0.05	0.04	kg CO_2/kWh$_e$
Fossil-Based H_2	Plant Life	Triangular	20	40	30	Years
	WACC	Triangular	4	10	4	%
	Economies of Scale Factor	Triangular	0.55	0.8	0.6	N/A
	Natural Gas GWP	Binomial	30	85	N/A	kg CO_2e/kg CH_4
	Natural Gas Leakage Rate	Uniform	0	4	N/A	%
	Natural Gas Processing Emissions	Triangular	0.1	0.5	0.3	kg CO_2/kg CH_4
	Natural Gas Price	Triangular	3	15	3	$/MMBTU
	CO_2 Transport and Storage Cost	Triangular	10	20	10	$/metric ton CO_2

Electricity- and Fossil-Based H_2	Grid Electricity Cost and Emissions	Multinomial	Cambium: Low RE Cost Mid-Case	Cambium: High RE Cost Mid-Case	Cambium: Mid-Case	\$/kWh_e each hour
	Grid Connection Capital Cost	Triangular	45	340	180	\$/kW_e
	Demand Charges	Triangular	0	30	10 (CA)<br>5 (NY, TX)	\$/max kW_e/month
	Life-Cycle Grid Emissions	Multinomial	Cambium: Low RE Cost Mid-Case	Cambium: High RE Cost Mid-Case	Cambium: Mid-Case	kg CO_2/kWh_e each hour
	Carbon Removal Cost	Triangular	100	600	100	\$/metric ton CO_2

8. Next Decade Technology Hydrogen Production Pathway Emissions and LCOH Figures

This section includes figures of the GHG emissions for each next-decade hydrogen production pathway explored in the study. It also includes a detailed cost breakdown figure for each hourly-reliable fossil-based production pathway in each state since this was not included in the main text.

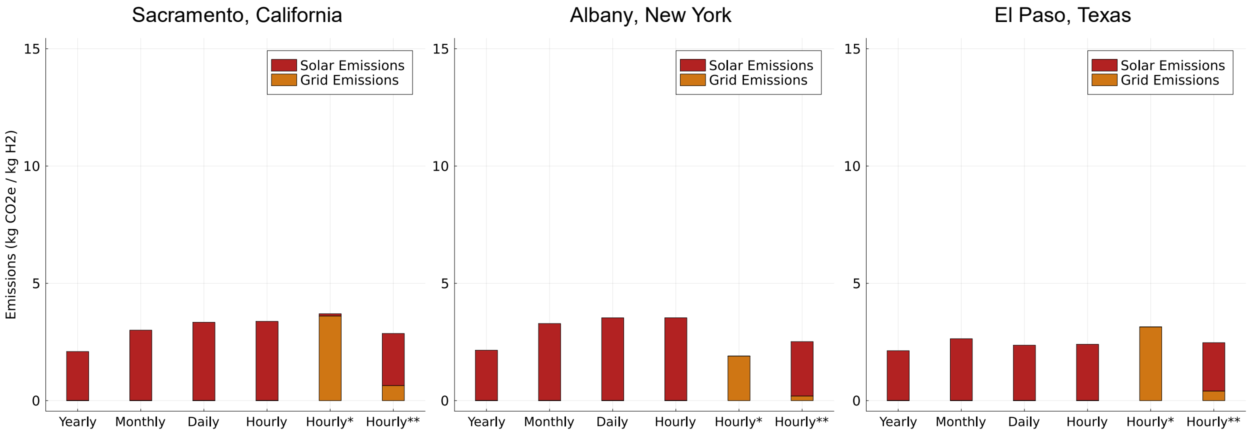

*Figure S.3: Next decade GHG emissions of solar PV-based hydrogen production under various levels of reliability. *Indicates an hourly-reliable production pathway that has an unconstrained grid connection for added reliability. ** Indicates and hourly-reliable production pathway that has a constrained grid connection (only 10% of the total electricity used at the facility can be from the grid) for added reliability.*

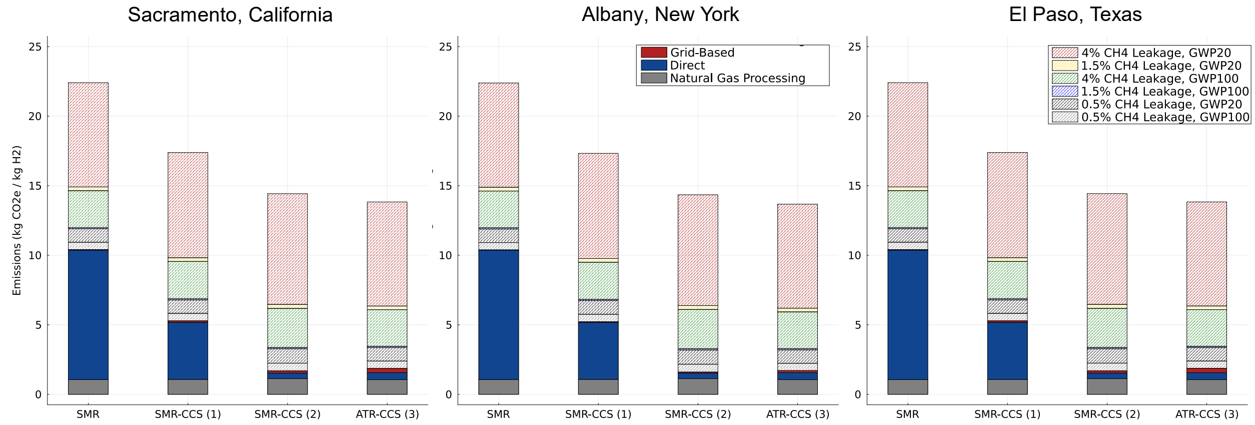

Figure S.4: Next-decade GHG emissions of various fossil-based hydrogen production pathways.

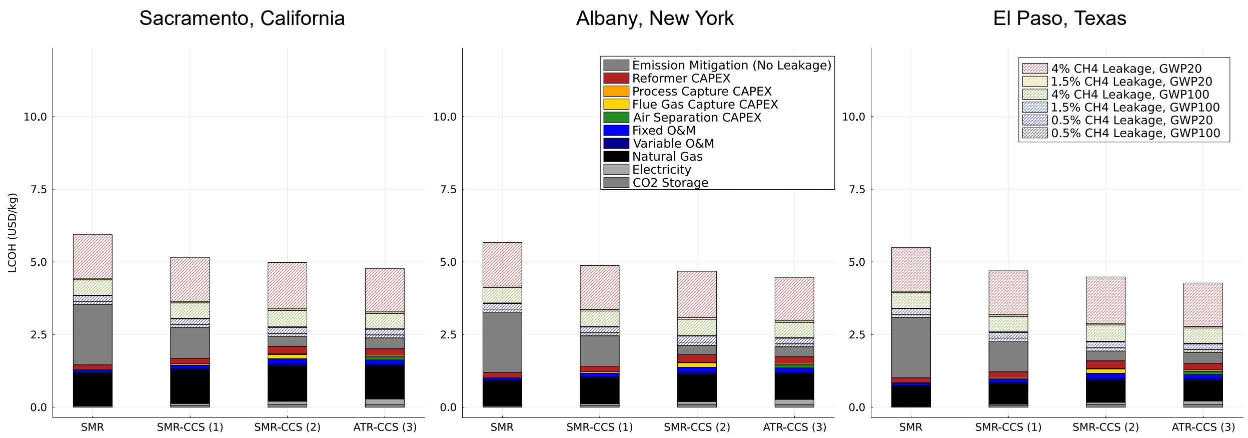

Figure S.5: Levelized cost of hourly-reliable net-zero hydrogen produced from fossil-based pathways. Pathways include SMR, SMR-CCS with process CO_2 capture (1), SMR-CCS with process and flue gas CO_2 capture (2), and ATR-CCS with process CO_2 capture (3).

9. Electricity-Based Hydrogen Production Pathway Operations Figures

This section contains tables and figures detailing system component sizing and electricity usage and curtailment for the electricity-based hydrogen production pathways under next-decade technology assumptions. Table S.10 contains system component sizes for each electricity-based pathway explored in this study as well as the percentage of system electricity that went unused at the production facility. Figures S.8 - S.22 show hourly and daily operations data specific to the California location.

Table S.10: Next-decade electricity-based system component sizing and electricity utilization for all pathways.

Location	Component	Yearly	Monthly	Daily	Hourly	Hourly*	Hourly**
Sacramento CA	Battery Storage (MWh_e)	0	0	0	0	584	684
	Hydrogen Storage (metric ton H_2)	0	2028	2332	2502	92	211
	Solar PV (MW_e)	1979	2842	3158	3195	94	2103
	Electrolyzer (MW_e)	1554	2200	2408	2406	590	1337
	Grid Connection (MW_e)	0	0	0	0	590	394
	Percent Electricity Unused (% system total)	2%	31%	38%	39%	1%	12%
Albany NY	Battery Storage (MWh_e)	0	0	6	0	1232	1203
	Hydrogen Storage (metric ton H_2)	0	2221	2411	2678	92	378
	Solar PV (MW_e)	2804	4288	4607	4610	0	3030
	Electrolyzer (MW_e)	1948	2901	2892	2887	577	1964
	Grid Connection (MW_e)	0	0	0	0	645	733
	Percent Electricity Unused (% system total)	5%	37%	41%	41%	1%	18%
El Paso TX	Battery Storage (MWh_e)	0	0	0	0	694	231
	Hydrogen Storage (metric ton H_2)	0	33	893	1047	173	314
	Solar PV (MW_e)	1870	2319	2076	2109	0	1815
	Electrolyzer (MW_e)	1492	1928	1763	1756	652	1487
	Grid Connection (MW_e)	0	0	0	0	694	231
	Percent Electricity Unused (% system total)	4%	22%	13%	14%	3%	9%

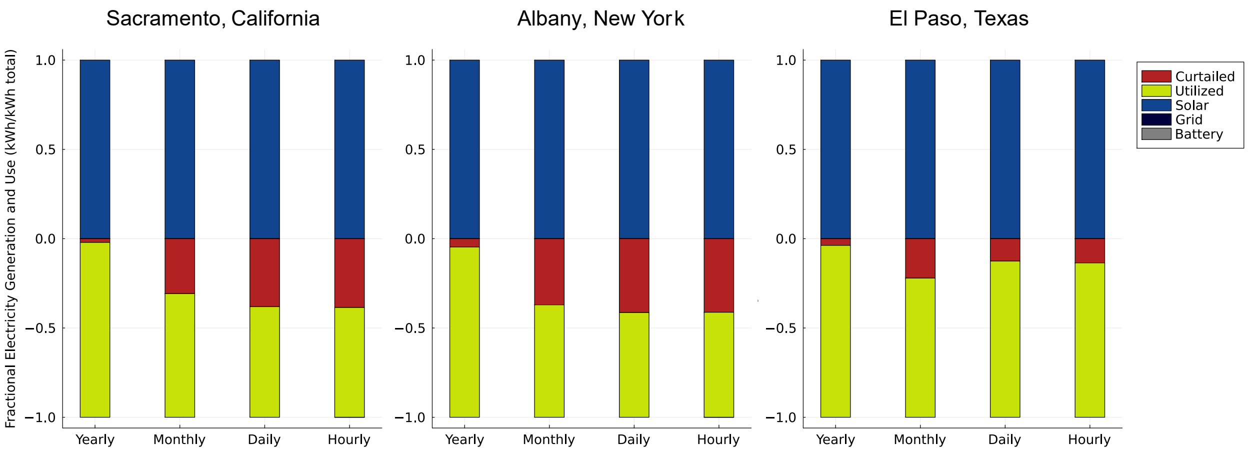

Figure S.6: Next decade electricity utilization for various levels of hydrogen production reliability pathways. Above the x-axis shows the fraction of electricity input from solar PV, the grid, or an onsite battery. Below the y-axis shows the fraction of the electricity input that is utilized or curtailed. Grid and battery electricity inputs are at or near zero in these pathways.

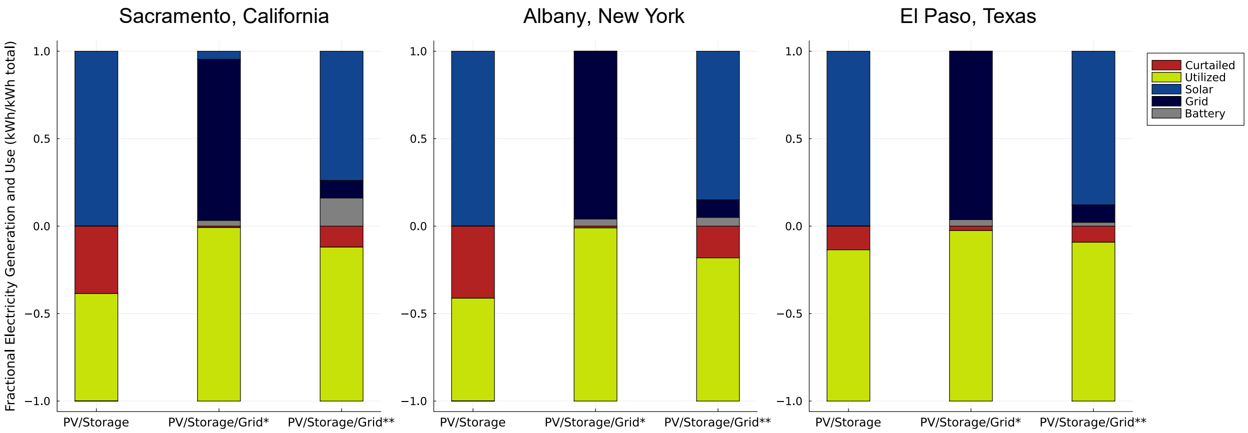

Figure S.7: Next decade electricity utilization for hourly production reliability pathways. Above the x-axis shows the fraction of electricity input from solar PV, the grid, or an onsite battery. Below the y-axis shows the fraction of the electricity input that is utilized or curtailed.

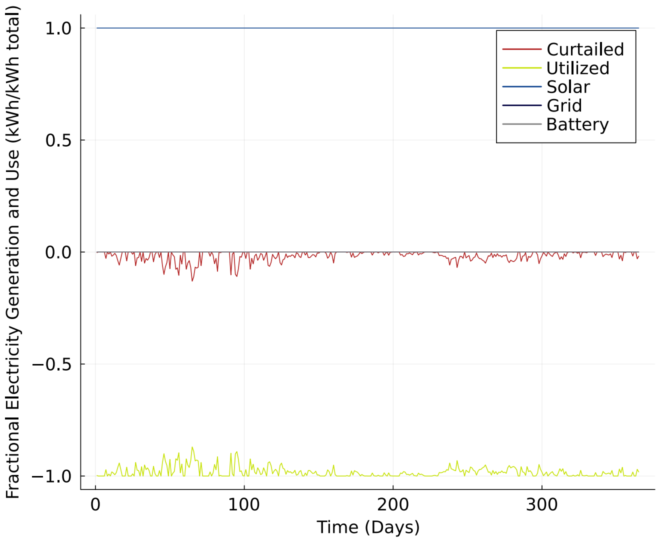

Figure S.8: Daily electricity utilization for the yearly reliable solar PV hydrogen production pathway in Sacramento, California. Above the x-axis shows the daily fraction of electricity input from solar PV, the grid, or an onsite battery. Below the y-axis shows the daily fraction of the electricity input that is utilized or curtailed. Grid and battery electricity inputs are at or near zero in this pathway.

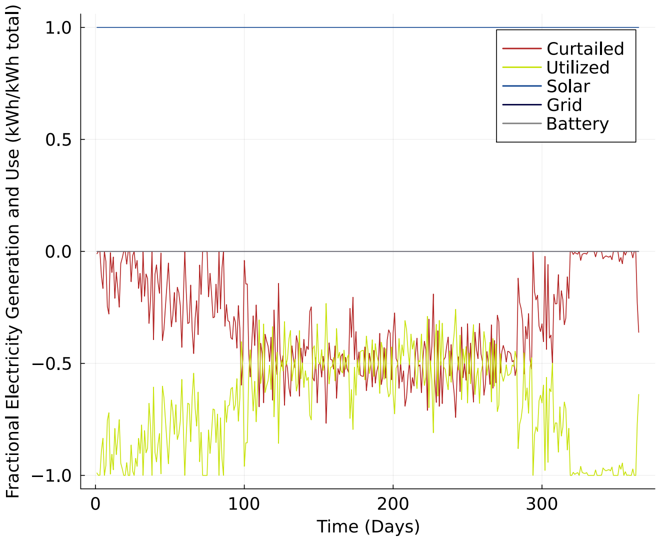

Figure S.9: Daily electricity utilization for the hourly reliable solar PV with storage hydrogen production pathway in Sacramento, California. Above the x-axis shows the daily fraction of electricity input from solar PV, the grid, or an onsite battery. Below the y-axis shows the daily fraction of the electricity input that is utilized or curtailed. Grid and battery electricity inputs are at or near zero in this pathway.

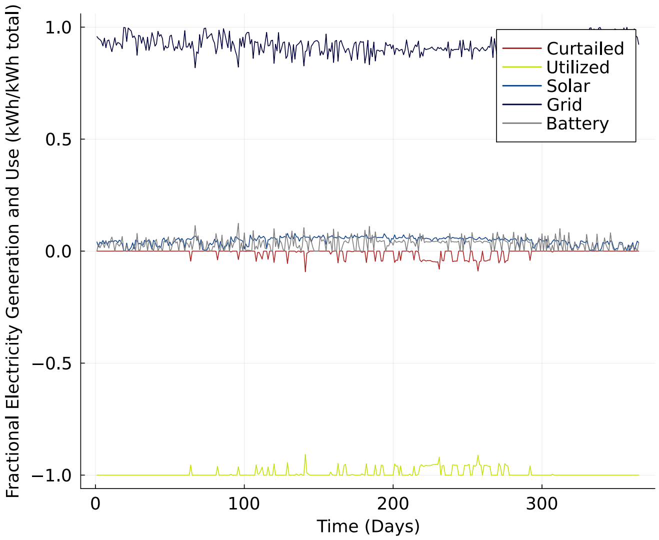

Figure S.10: Daily electricity utilization for the hourly reliable solar PV, storage, and grid connected hydrogen production pathway in Sacramento, California (Equivalent to PV/Storage/Grid pathway). Above the x-axis shows the daily fraction of electricity input from solar PV, the grid, or an onsite battery. Below the y-axis shows the daily fraction of the electricity input that is utilized or curtailed.*

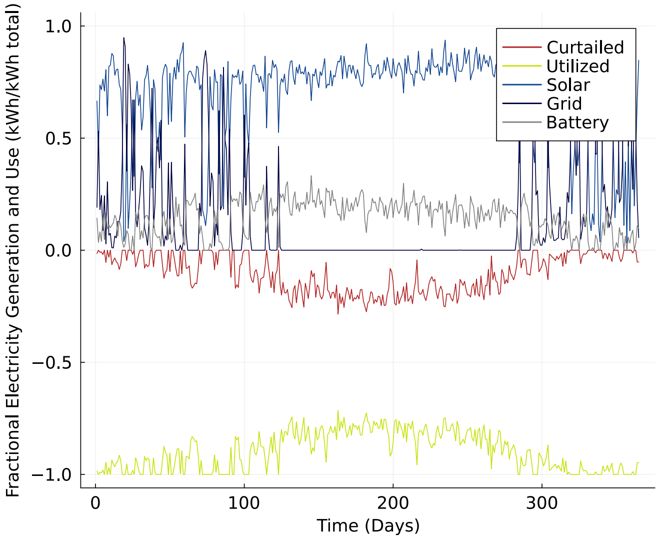

*Figure S.11: Daily electricity utilization for the hourly reliable solar PV, storage, and limited grid connected hydrogen production pathway in Sacramento, California (Equivalent to PV/Storage/Grid** pathway). Above the x-axis shows the daily fraction of electricity input from solar PV, the grid, or an onsite battery. Below the y-axis shows the daily fraction of the electricity input that is utilized or curtailed.*

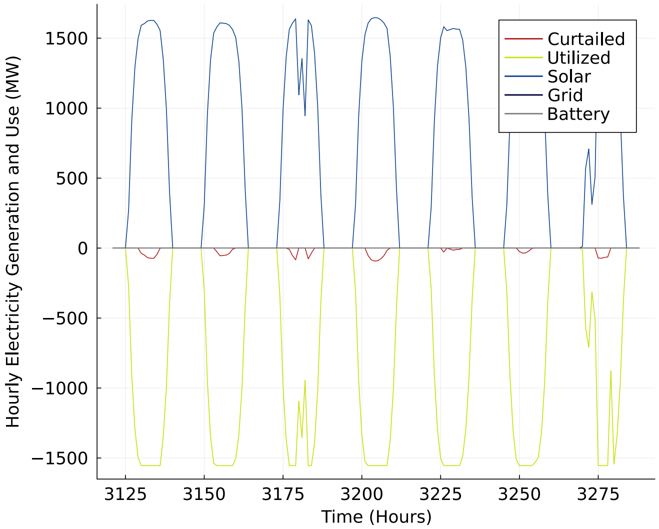

Figure S.12: Hourly electricity utilization for the yearly reliable solar PV hydrogen production pathway for a week in the month of May in Sacramento, California. Above the x-axis shows the hourly electricity input from solar PV, the grid, or an onsite battery. Below the y-axis shows the hourly electricity input that is utilized or curtailed. Grid and battery electricity inputs are at or near zero in this pathway.

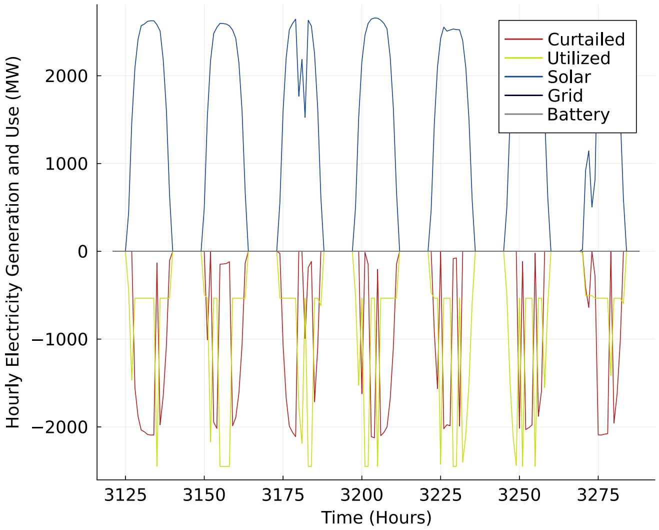

Figure S.13: Hourly electricity utilization for the hourly reliable solar PV and storage hydrogen production pathway for a week in the month of May in Sacramento, California (Equivalent to PV/Storage pathway). Above the x-axis shows the hourly electricity input from solar PV, the grid, or an onsite battery. Below the y-axis shows the hourly electricity input that is utilized or curtailed. Grid and battery electricity inputs are at or near zero in this pathway.

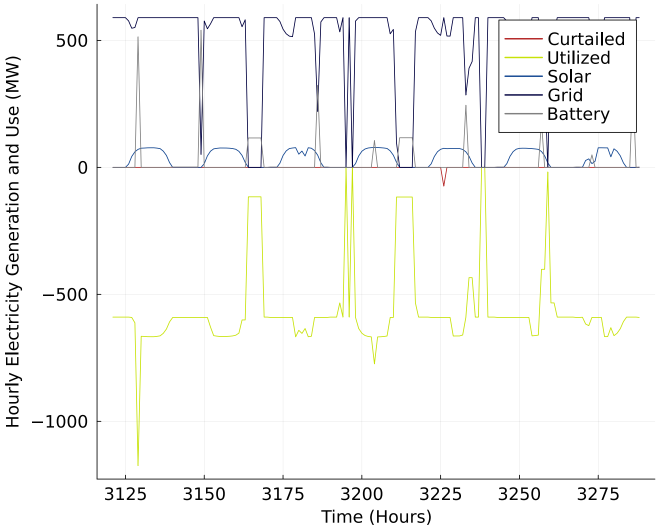

Figure S.14: Hourly electricity utilization for the hourly reliable solar PV, storage, and grid connected hydrogen production pathway for a week in the month of May in Sacramento, California (Equivalent to PV/Storage/Grid pathway). Above the x-axis shows the hourly electricity input from solar PV, the grid, or an onsite battery. Below the y-axis shows the hourly electricity input that is utilized or curtailed.*

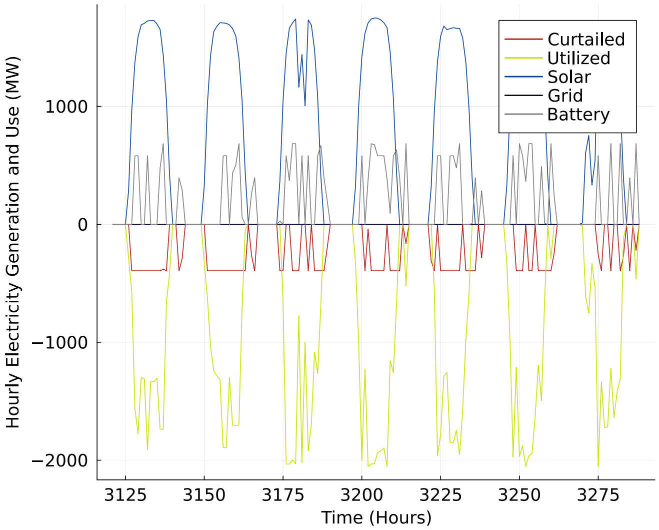

*Figure S.15: Hourly electricity utilization for the hourly reliable solar PV, storage, and limited grid connected hydrogen production pathway for a week in the month of May in Sacramento, California (Equivalent to PV/Storage/Grid** pathway). Above the x-axis shows the hourly electricity input from solar PV, the grid, or an onsite battery. Below the y-axis shows the hourly electricity input that is utilized or curtailed.*

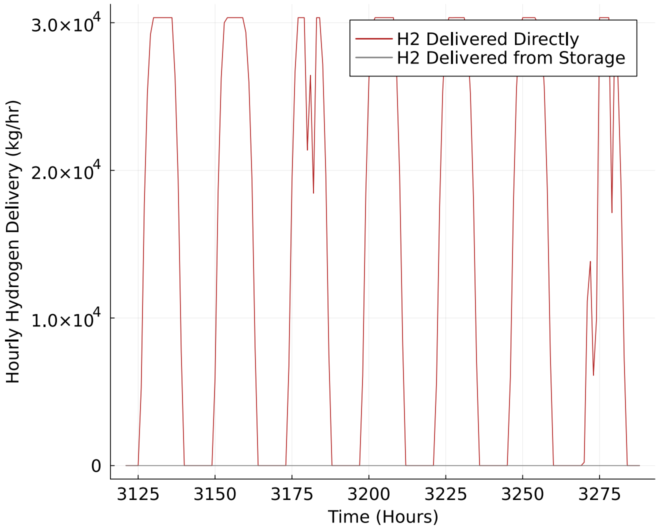

Figure S.16: Hourly hydrogen delivery for yearly reliable solar PV hydrogen production for a week in the month of May in Sacramento, California.

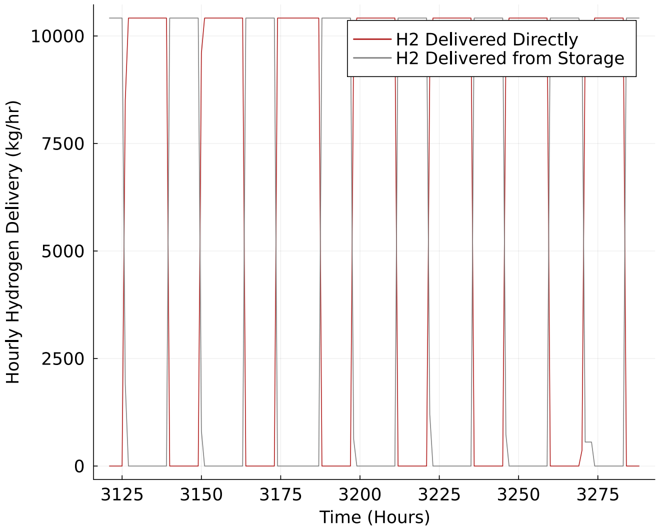

Figure S.17: Hourly hydrogen delivery for hourly reliable solar PV and storage hydrogen production pathway for a week in the month of May in Sacramento, California (Equivalent to PV/Storage pathway).

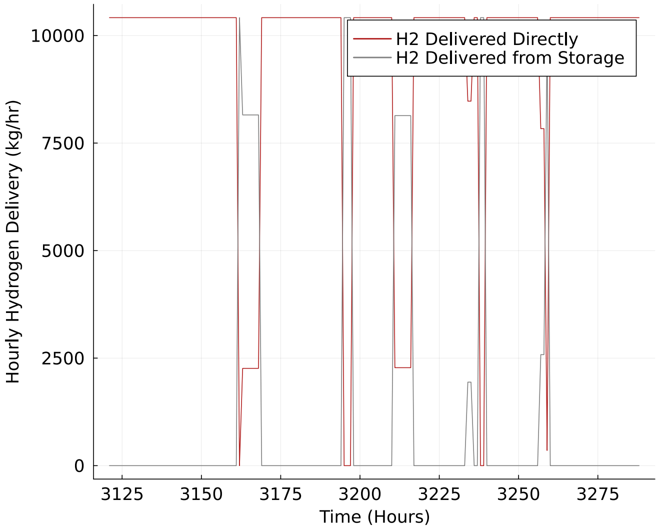

Figure S.18: Hourly hydrogen delivery for hourly reliable solar PV, storage, and grid connected hydrogen production pathway for a week in the month of May in Sacramento, California (Equivalent to PV/Storage/Grid pathway).*

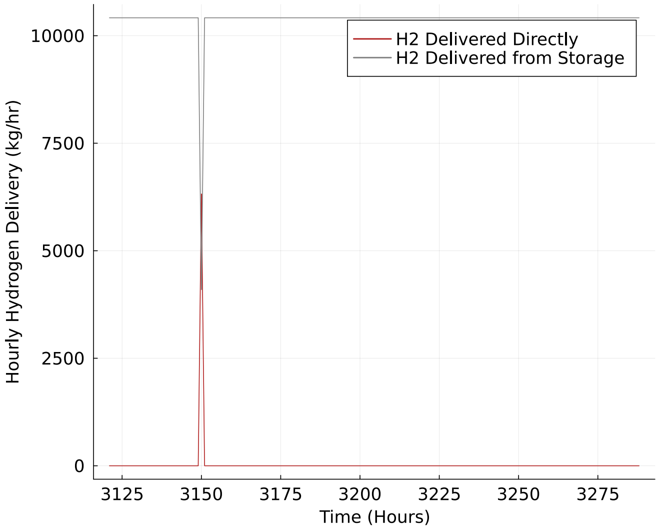

*Figure S.19: Hourly hydrogen delivery for hourly reliable solar PV, storage, and limited grid connected hydrogen production pathway for a week in the month of May in Sacramento, California (Equivalent to PV/Storage/Grid** pathway).*

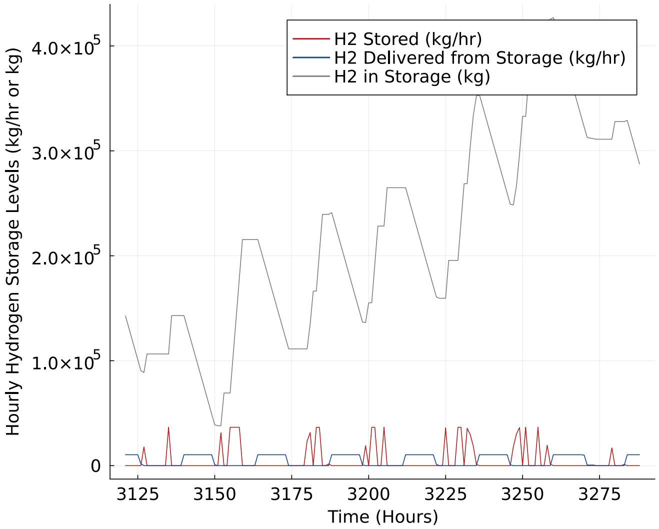

Figure S.20: Hourly hydrogen storage for hourly reliable solar PV and storage hydrogen production pathway for a week in the month of May in Sacramento, California (Equivalent to the PV/Storage pathway).

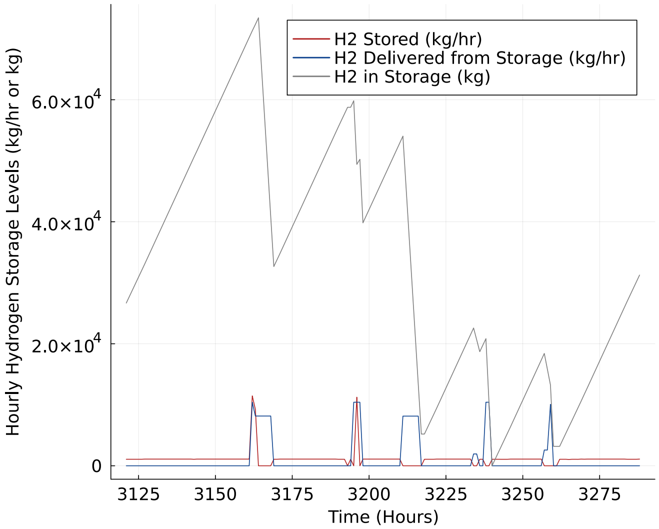

Figure S.21: Hourly hydrogen storage for hourly reliable solar PV, storage, and grid connected hydrogen production pathway for a week in the month of May in Sacramento, California (Equivalent to the PV/Storage/Grid pathway).*

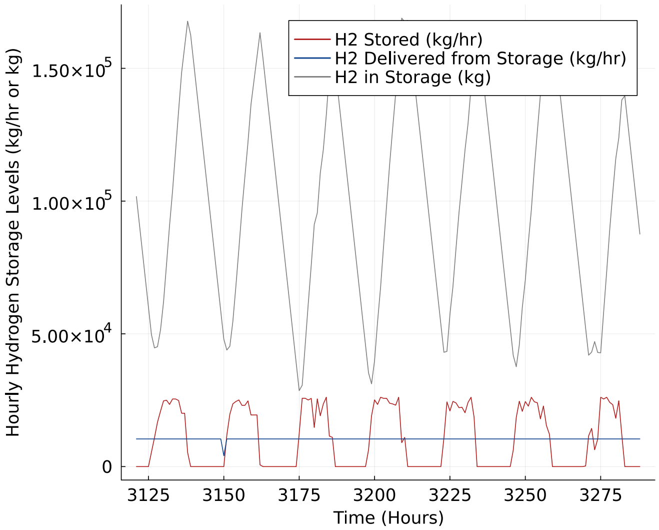

*Figure S.22: Hourly hydrogen storage for hourly reliable solar PV, storage, and limited grid connected hydrogen production pathway for a week in the month of May in Sacramento, California (Equivalent to the PV/Storage/Grid** pathway).*

10. LCOH Figures with Current and Mid-Century Timeframes

This section contains LCOH figures found in the main text (Figures 3 and 4) but using current, next-decade, and mid-century technology input assumptions. We find that all hourly-reliable, net-zero hydrogen production costs fall significantly by mid-century in comparison to current costs.

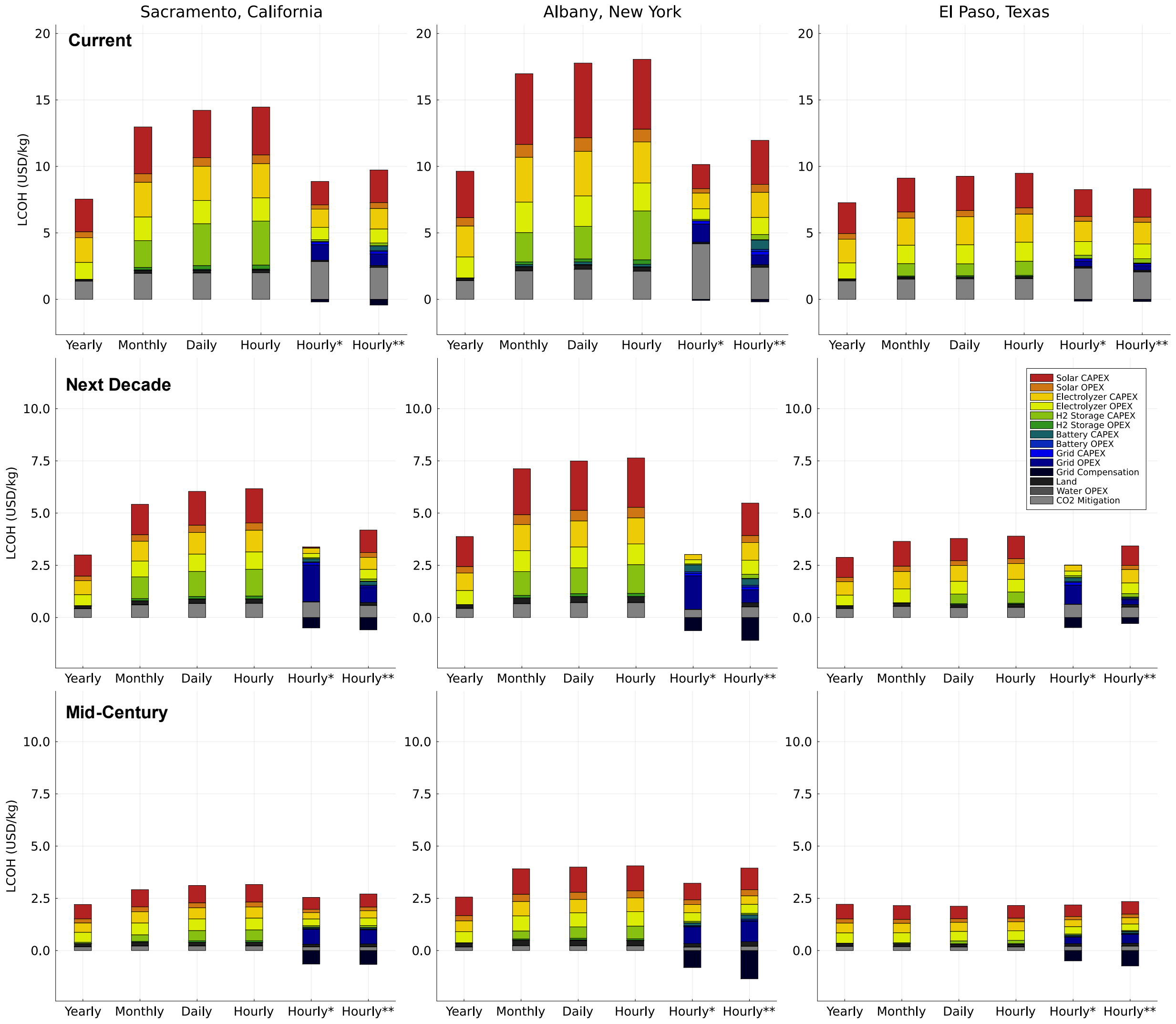

Figure S.23: Net-zero hydrogen production cost comparison between all electricity-based production pathways looking at all timeframes and locations.

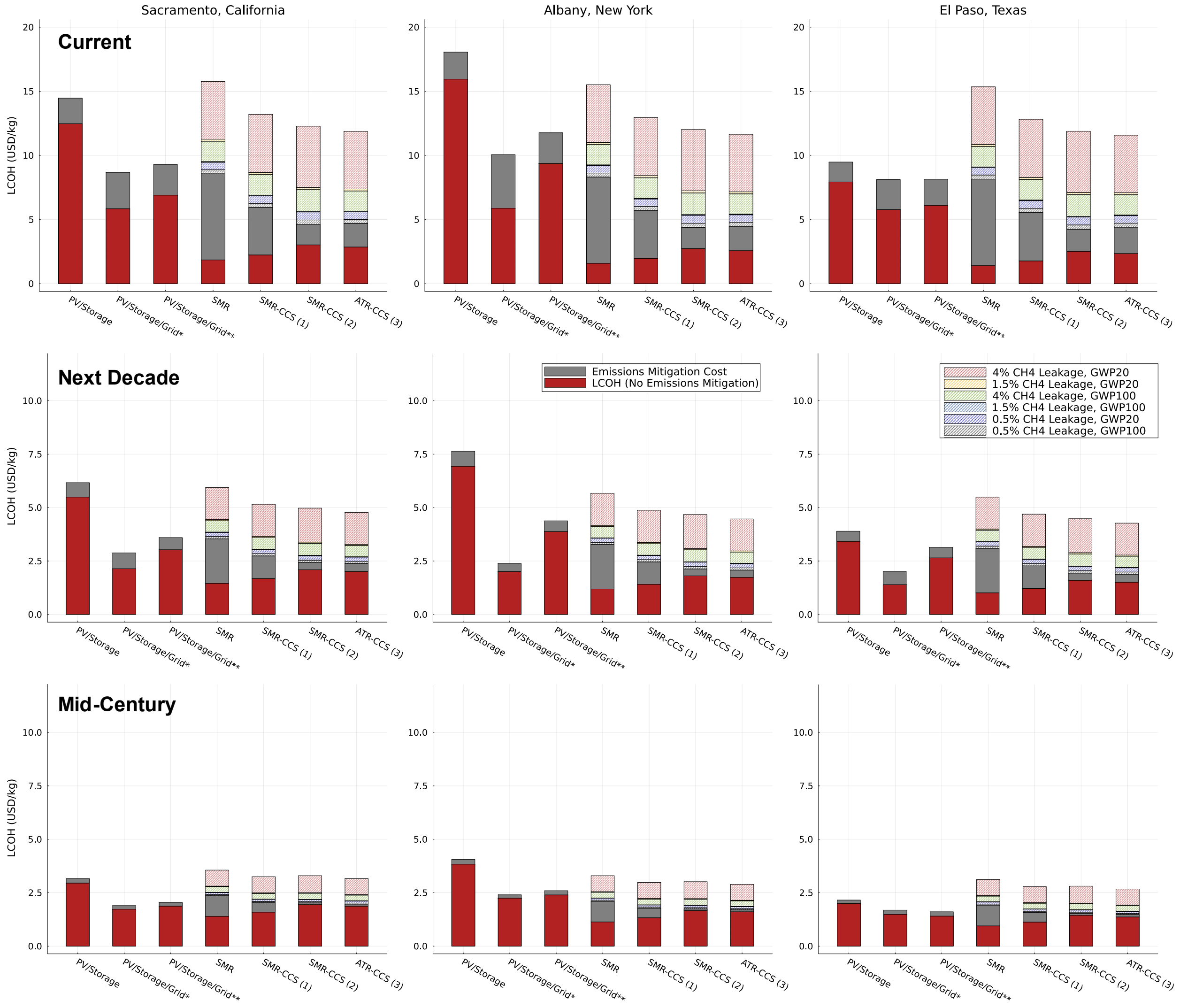

Figure S.24: Hourly reliable, net-zero hydrogen production cost comparison between electricity-based and fossil-based production pathways looking at all timeframes and locations.

11. Inflation Reduction Act Analysis with Current Technology Timeframe

This section contains a version of Figure 6 from the main text, but with current technology assumptions. As shown, even with embodied emissions excluded, the electricity-based pathways do not reach cost parity with fossil-based alternatives when tax credits from the Inflation Reduction Act are considered. This remains true for all locations explored in the study under the current technology assumption.

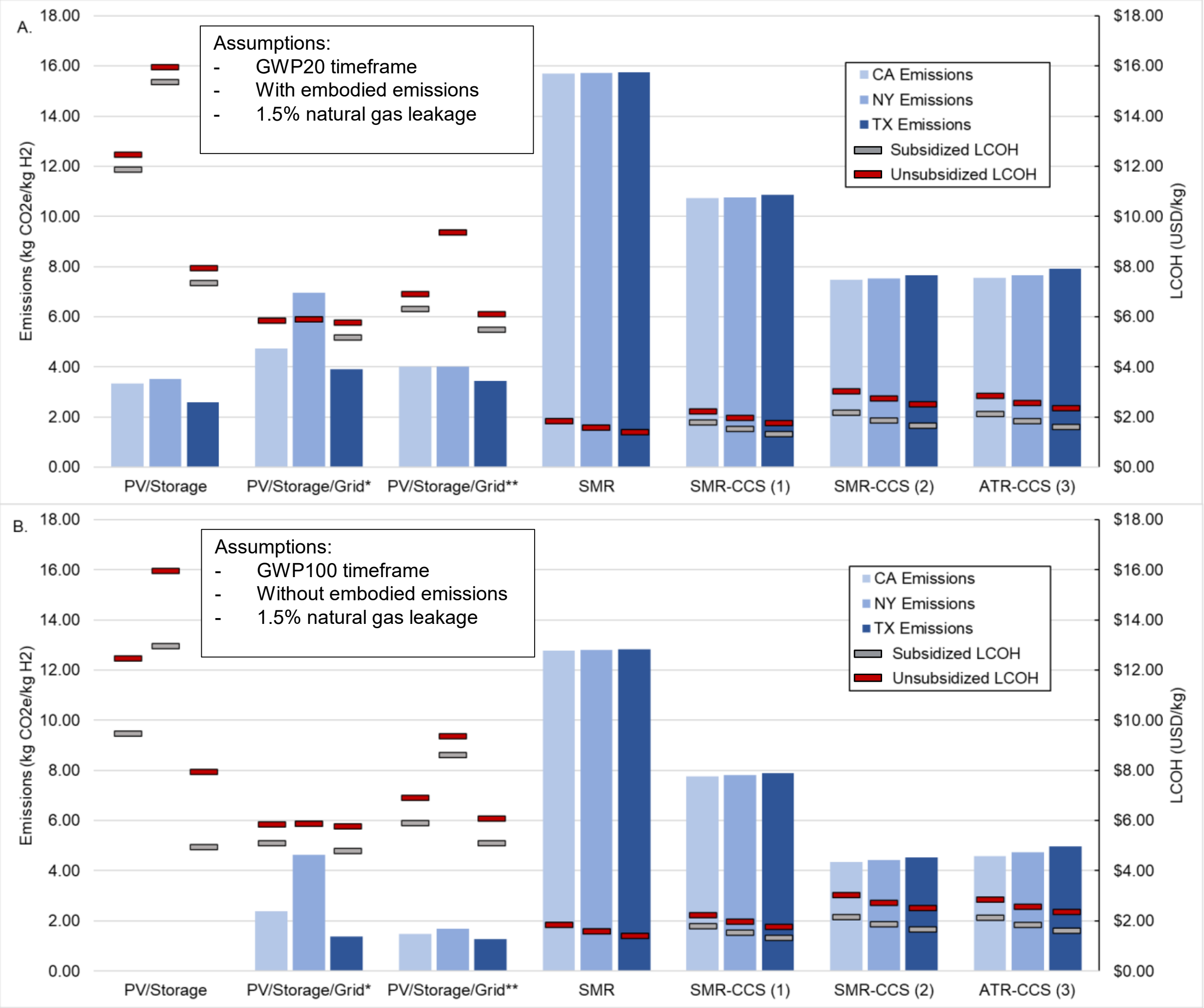

Figure S.25: Current technology IRA emission and cost analysis. (a) Total GHG emissions (in kg CO_2e / kg H_2 produced), unsubsidized LCOH (in \$/kg H_2), and subsidized LCOH (in \$/kg H_2) for each hourly reliable hydrogen production pathway, for each state, assuming a 20-year GWP timeframe, 1.5% natural gas leakage, and including embodied emissions of electricity-generating sources. (b) Total GHG emissions (in kg CO_2e / kg H_2 produced), unsubsidized LCOH (in \$/kg H_2), and subsidized LCOH (in \$/kg H_2) for each hourly reliable hydrogen production pathway, for each state, assuming a 100-year GWP timeframe, 1.5% natural gas leakage, and excluding embodied emissions. The subsidized LCOH values that consider 45V are valid for the first 10 years of project operation as defined in the IRA. The subsidized LCOH values that consider 45Q are valid for the first 12 years of project operation as defined in the IRA.

References

[1] U.S. Bureau of Labor Statistics, “CPI Inflation Calculator,” 2023. https://www.bls.gov/data/inflation_calculator.htm.

[2] National Renewable Energy Laboratory, “System Advisor Model,” 2020. https://sam.nrel.gov/ (accessed Sep. 29, 2022).

[3] E. D. Sherwin, “Electrofuel Synthesis from Variable Renewable Electricity: An Optimization-Based Techno-Economic Analysis,” *Environ. Sci. Technol.*, vol. 55, no. 11, pp. 7583–7594, Jun. 2021, doi: 10.1021/ACS.EST.0C07955/SUPPL_FILE/ES0C07955_SI_001.PDF.

[4] Hydrogen Tools, “Lower and Higher Heating Values of Fuels,” 2023. https://h2tools.org/hyarc/calculator-tools/lower-and-higher-heating-values-fuels.

[5] National Renewable Energy Laboratory, “H2A-Lite: Hydrogen Analysis Lite Production Model,” 2022. https://www.nrel.gov/hydrogen/h2a-lite.html (accessed May 04, 2023).

[6] Y. Acevedo, J. Huya-Kouadio, K. McNamara, J. Prosser, and B. James, “Comparative Study of Levelized Cost of Hydrogen for Alkaline, PEM, AEM, and SOE Electrolyzer Plants,” 2023.

[7] M. Bolinger, R. Wiser, and E. O’Shaughnessy, “Levelized cost-based learning analysis of utility-scale wind and solar in the United States,” *Iscience*, 2022, Accessed: Nov. 22, 2022. [Online]. Available: https://www.sciencedirect.com/science/article/pii/S2589004222006496.

[8] National Renewable Energy Laboratory, “Annual Technology Baseline,” 2020. https://atb.nrel.gov/ (accessed May 02, 2023).

[9] J. Ramsden, T.; Kroposki, B.; Levene, “Opportunities for Hydrogen-Based Energy Storage for Electric Utilities. Golden,” pp. 1–17, 2008, [Online]. Available: nha.confex.com/nha/2008/recordingredirect.cgi/id/352.

[10] W. Cole, A. W. Frazier, and C. Augustine, “Cost Projections for Utility-Scale Battery Storage: 2021 Update,” 2021. Accessed: Apr. 22, 2023. [Online]. Available: www.nrel.gov/publications.

[11] W. Colella, B. D. James, J. M. Moton, G. Saur, and T. Ramsden, “Techno-economic Analysis of PEM Electrolysis for Hydrogen Production,” Golden, Colorado, Feb. 2014.

[12] Solar Land Lease, “Lease Rates for Solar Farms: How Valuable Is My Land?” https://www.solarlandlease.com/lease-rates-for-solar-farms-how-valuable-is-my-land (accessed May 28, 2021).

[13] National Renewable Energy Laboratory, “Distributed Generation Renewable Energy Estimate of Costs,” 2016. https://www.nrel.gov/analysis/tech-lcoe-re-cost-est.html (accessed May 28, 2021).

[14] P. Gagnon, B. Cowiestoll, and M. Schwarz, “Cambium 2022 Data,” 2023. [Online]. Available: https://scenarioviewer.nrel.gov/.

[15] O. Edenhofer *et al.*, “Renewable energy sources and climate change mitigation: Special report of the intergovernmental panel on climate change,” Cambridge University Press, 2012. doi: 10.1017/CBO9781139151153.

[16] United Nations, “Life Cycle Assessment of Electricity Generation Options,” 2021.

[17] National Renewable Energy Laboratory, “Identifying Potential Markets for Behind-the-Meter Battery Energy Storage: A Survey of U.S. Demand Charges,” 2017.

[18] National Renewable Energy Laboratory, “Energy Analysis: Life Cycle Assessment Harmonization,” 2012. https://www.nrel.gov/analysis/life-cycle-assessment.html (accessed Sep. 29, 2022).

[19] J. Gertner, “The Tiny Swiss Company That Thinks It Can Help Stop Climate Change,” *The New York Times*, 2019.

[20] National Energy Technology Laboratory, “Comparison of Commercial, State-of-the-Art, Fossil-Based Hydrogen Production Technologies,” 2022. [Online]. Available: https://www.osti.gov/servlets/purl/1862910/.

[21] J. Theis, “Cost Estimation Methodology for NETL Assessments of Power Plant Performance,” 2019. Accessed: Jan. 18, 2022. [Online]. Available: www.netl.doe.gov.

[22] U.S. Energy Information Administration, “Annual Energy Outlook 2023,” 2023. https://www.eia.gov/outlooks/aeo/ (accessed May 09, 2023).

[23] National Renewable Energy Laboratory, “H2A: Hydrogen Analysis Production Models,” 2018. https://www.nrel.gov/hydrogen/h2a-production-models.html (accessed Dec. 08, 2021).

[24] C. E. Clark, J. Han, A. Burnham, J. B. Dunn, and M. Wang, “Life-Cycle Analysis of Shale Gas and Natural Gas,” 2011. [Online]. Available: https://publications.anl.gov/anlpubs/2012/01/72060.pdf.

[25] J. De Chalendar, “Tracking emissions in the US electricity system,” *Stanford University*, 2022. https://gridemissions.jdechalendar.su.domains/#/ (accessed Sep. 29, 2022).

[26] Stanford University and Energy Futures Initiative, “An Action Plan for Carbon Capture and Storage in California: Opportunities, Challenges, and Solutions,” 2020. [Online]. Available: www.energy.stanford.edu.

[27] U.S. Environmental Protection Agency, “Understanding Global Warming Potentials,” 2022. https://www.epa.gov/ghgemissions/understanding-global-warming-potentials (accessed Oct. 06, 2022).

[28] R. W. Howarth and M. Z. Jacobson, “How green is blue hydrogen?,” *Energy Sci. Eng.*, vol. 9, no. 10, pp. 1676–1687, Oct. 2021, doi: 10.1002/ESE3.956.

[29] International Energy Agency, “Natural gas prices in Europe, Asia and the United States, Jan 2020-February 2022,” 2022. https://www.iea.org/data-and-statistics/charts/natural-gas-prices-in-europe-asia-and-the-united-states-jan-2020-february-2022.

[30] J. S. Rutherford *et al.*, “Closing the methane gap in US oil and natural gas production emissions inventories,” *Nat. Commun.*, Aug. 2021, doi: 10.1038/s41467-021-25017-4.

[31] Y. Chen *et al.*, “Quantifying Regional Methane Emissions in the New Mexico Permian Basin with a Comprehensive Aerial Survey,” *Environ. Sci. Technol.*, vol. 56, no. 7, pp. 4317–4323, 2022, doi: 10.1021/acs.est.1c06458.
